# Cystic echinococcosis (*Echinococcus granulosus* *sensu lato* infection) in Tunisia, a One Health perspective for a future control programme[Fn FN1]

**DOI:** 10.1051/parasite/2024029

**Published:** 2024-06-13

**Authors:** Mohamed Gharbi, Patrick Giraudoux

**Affiliations:** 1 Laboratory of parasitology, Université de la Manouba. National School of Veterinary Medicine of Sidi Thabet 2020 Sidi Thabet Tunisia; 2 Chrono-environnement, Université de Franche-Comté/CNRS, La Bouloie 25030 Besançon France

**Keywords:** *Echinococcus granulosus*, Cestode, Slaughterhouse, Control, Constraint, One Health, Tunisia

## Abstract

The emergence of pandemics with dramatic consequences for human health has obscured endemic diseases that continue to pose a problem for human and animal health in several regions of the world. Among these diseases, cystic echinococcosis, a zoonotic parasitic infection caused by a group of cestodes, *Echinococcus granulosus sensu lato*, remains a real human and animal health problem in several regions of the world, including the Mediterranean Basin. Despite the implementation of a number of governmental control programmes using several tools (dog treatment, meat inspection, etc.), this infection is still highly prevalent in North Africa. Here we present a review of the epidemiology of cystic echinococcosis in Tunisia, an analysis of the constraints limiting the effectiveness of the control programmes implemented, and finally argue for the use of the One Health framework to improve the effectiveness of future programmes.

## Abstract (fr)

L’émergence de pandémies affectant de manière très importante la santé humaine a occulté les maladies endémiques qui continuent de représenter un problème de santé animale et humaine dans plusieurs régions du monde. Parmi ces maladies, l’échinococcose kystique, une parasitose zoonotique provoquée par un groupe de cestodes, *Echinococcus granulosus sensu lato,* reste un réel problème de santé humaine et animale dans plusieurs régions du monde, incluant le Bassin méditerranéen. Malgré la mise en place de nombreux programmes de lutte utilisant plusieurs outils (traitement des chiens, inspection des viandes, etc.), cette infection est encore hautement prévalente en Afrique du Nord. Nous présentons ici une revue de l’épidémiologie de l’échinococcose kystique en Tunisie, une analyse des contraintes limitant l’efficacité des programmes de lutte mis en place, et finalement nous argumentons l’utilisation du concept « Une seule santé » pour améliorer l’efficacité des programmes futurs.

## Abstract (ar)

أدى ظهور الأوبئة التي تؤثر بشكل كبير على صحة الإنسان إلى حجب الأمراض المستوطنة التي لا تزال تمثل مشكلة لصحة الحيوان والإنسان في العديد من مناطق العالم. ومن بين هذه الأمراض، لا يزال مرض الكيس المائي، مرض طفيلي مشترك المتسبب فيه مجموعة من الديدان الشريطية، المشوكة الحبيبية بالمعنى الواسع Echinococcus granulosus sensu lato ، يمثل مشكلة حقيقية لصحة الانسان و الحيوان في العديد من مناطق العالم، بما في ذلك حوض البحر الأبيض المتوسط. وعلى الرغم من تنفيذ عدد من برامج المكافحة باستخدام عدة أدوات (علاج الكلاب، فحص اللحوم، وغيرها)، إلا أن هذه العدوى لا تزال منتشرة بشكل كبير في شمال أفريقيا. نقدم هنا استعراضا لوبائيات مرض الكيس المائي في تونس، وتحليلا للقيود التي تحد من فعالية برامج المكافحة المطبقة، وأخيرا نناقش استخدام مفهوم الصحة الواحدة لتحسين فعالية البرامج المستقبلية.

## Introduction

A large number of infectious diseases have emerged in all regions of the world due to various ecological and politico-economic changes, as well as intensive movements of people and animals. Approximately 70% of these diseases are zoonotic, and a number pose major challenges to decision makers in the human and animal health sectors (COVID-19, Crimean Congo haemorrhagic fever, West Nile virus disease, Rift valley fever, Avian flu, Nipah virus disease, etc.). With each outbreak, global mobilisation of all stakeholders including international organisations (WHO, WOAH and FAO), and national and regional human and animal health services made it possible to limit the impact of these emerging pathogens and in most cases, to effectively control their spread. These “great fears” mobilised attention, manpower and resources that were lacking for “old”, neglected, endemic diseases, such as tuberculosis and parasitic diseases: geohelminthioses and leishmanioses. In Tunisia, several studies have demonstrated the role of dogs in maintaining the epidemiological cycles of several zoonotic diseases, mainly rabies [8], visceral leishmaniosis (*Leishmania infantum* infection) [3], spotted Mediterranean fever (*Rickettsia conorii* infection) [23] and cystic echinococcosis (CE) (*Echinococcus granulosus sensu lato* infection) [30]. Although the costs and impacts of these diseases have not been quantified for all of them, it is reasonable to assume that they are of varying importance to human and animal health and to the economy. The epidemiology of certain other parasitic animal diseases is not well studied in Tunisia, such as those caused by *Toxocara* spp. and *Giardia intestinalis* infections. Finally, other zoonotic infections are of minor importance such as tick infestation (*Rhipicephalus sanguineus*), infection by *Dipylidium caninum* and flea infestations. Cystic echinococcosis is the official name of *Echinococcus granulosus sensu lato* infection as recommended by the International consensus on terminology to be used in the field of echinococcosis and that will be adopted here [39]. CE continues to cause high morbidity in humans and significant economic losses due to animal infections [9, 37], mainly in the Maghreb region where it is endemic [4, 16, 19]. Control programmes targeting CE have not been effective for many decades, despite substantial human and financial resources.

Worldwide, with the exception of a few islands, no programme based on slaughterhouse inspection and dog control and purgation, and sometimes sheep vaccination, has reached CE elimination, even over a long period of several decades [13]. In fact, elimination on a continent is much more complicated because movement of the parasite’s definitive and intermediate hosts is much more difficult to restrict, while community perceptions, traditions and governance are more spatially varied, and the species richness of wildlife, with more hosts to act as reservoirs for the parasite, is greater [17]. CE endemicity is spatially heterogeneous across several space-time scales and it may be affected by global environmental changes. Although the One Health concept is not new and has been at the forefront of interdisciplinary and multisectoral discussions for more than 20 years, there is now increased interest in applying and translating this holistic approach into actions [1], especially in complex transmission systems where human and animal health are jointly affected and driven by a range of ecological factors. The aim of this paper is to review the constraints of CE control in Tunisia and to recommend control strategies in the light of the One Health concept.

## Cystic echinococcosis transmission in Tunisia

CE is a zoonotic helminthosis caused by the presence and development of *E. granulosus* metacestodes in intermediate hosts (herbivores and omnivores). Five species have been described in Africa with different hosts and life cycles, and varying epidemiological importance locally [14]. Little is known about these genotypes in Tunisia, but based on comparison with neighbouring countries, one can assume that *Echinococcus granulosus sensu stricto* is the main species circulating, with possibly *E. canadensis* G6/G7 – taeniid in camels and *E. ortleppi* in cattle in addition. However, none of them are completely specific and can infect various intermediate host species to some degree. The adult forms are small taeniid worms and they develop in the midgut of canid definitive hosts. Dogs play an important role in transmission for two reasons: (i) the number of dogs is by far the highest of all canid species in Tunisia, and (ii) there is close contact between dogs and several intermediate hosts (mainly small ruminants including sheep and goats, and secondly, cattle and other domestic ungulates). This contact is both intense and frequent because dogs, mainly shepherd dogs, and small ruminants are together all the time.

Most data about CE are old and fragmentary, as in other endemic countries, and CE in Tunisia is important for four reasons:

- Importance in human health: in 1997, the surgical prevalence of CE in Tunisia was estimated at 15/100,000 inhabitants based on data from public hospitals [20]. Many other cases may not have been identified at that time (e.g., asymptomatic cases, people operated in private hospitals, etc.) and have not been updated since, so the number remains a large underestimate. A sero-epidemiological survey carried out in Tunisia, revealed, as expected, a large discrepancy between the surgical prevalence and seroprevalence of CE in humans. The latter was estimated to be between 1.4% and 4%, corresponding to 1,500–4,000 seropositive persons/100,000 [21]. In addition, unusual locations of cysts (e.g., the heart and brain) can increase the severity of the disease and make diagnosis more difficult in some cases [12, 32].
- Importance in animal health: the presence of cysts in vital organs, mainly the lungs and the liver can lead to high intensity of infection in some animals, so high that it affects their general health status leading people to cull them (Figure 1). In these animals, CE is often included in the differential diagnosis of “skinny ewe syndrome”. However, most infected animals with low levels of infection are asymptomatic.
  **Figure 1:**
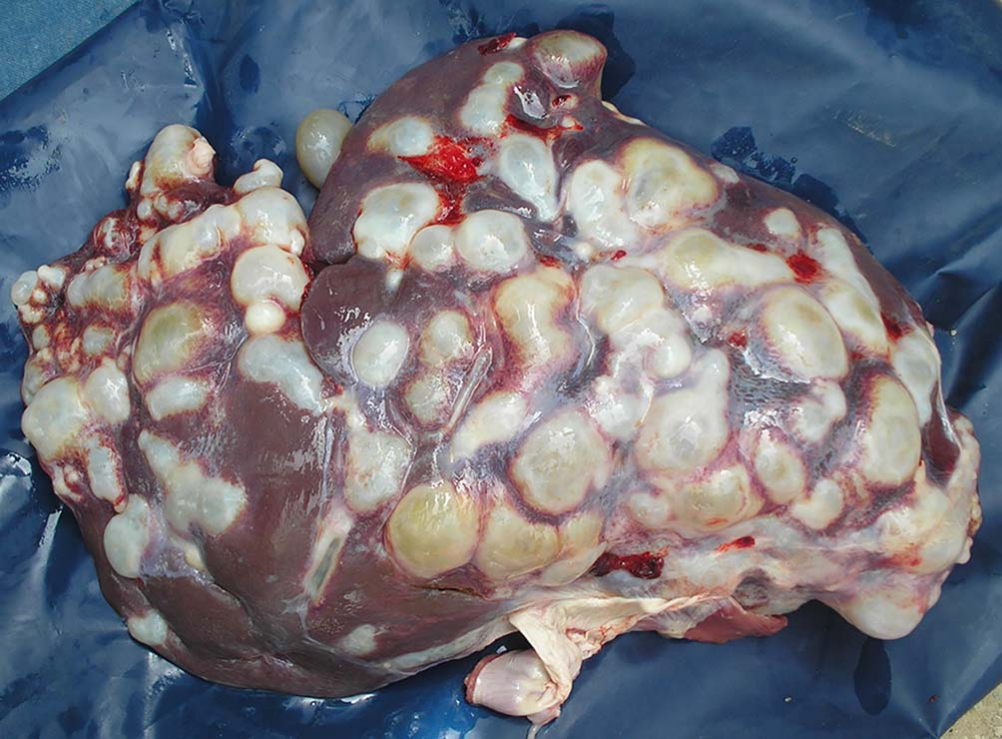
A heavily infected ewe liver, called “egg basket” locally.
- Economic importance: Majorowsky *et al.* [30] estimated the cost of CE in Tunisia to be between US$ 10 and 19 million/year, representing 44% and 56% of losses due to human and animal infections, respectively. To these costs, can be added others not considered in this article, such as the indirect effects of infection on animal production, including weight loss, hypogalaxia, mortality and culling. All these losses are high, because they are caused by asymptomatic infections, are persistent and affect a large (but unknown) proportion of the animal population.
- Impact on wild animals: although the presence of a wild cycle of *E. granulosus* has not been established, wild carnivores (hyenas, wolfs, jackals and foxes) are definitive hosts of the parasite in Tunisia. On the other hand, large wild ruminants like the antelope addax (*Addax nasomaculatus*) [7] and wild boars (*Sus scrofa*) [27] are intermediate hosts. The high prevalence of infection in wild boars (56/297; 18.9%, 95% CI: 14.7–23.9%) [27] could be explained by the presence of boars at night near human habitations in several regions of Tunisia, where non-treated dogs are present.

## Cystic echinococcosis control options in the Tunisian context

### Constraints for controlling cystic echinococcosis in the Tunisian context

Despite the implementation of several control programmes against CE in Tunisia, infection rates remain high in different species including humans, other intermediate hosts and dogs. A survey conducted in different regions showed that the prevalence of *E. granulosus* in dogs ranged from 8.3% to 41.3% [11]. A more recent study in southern Tunisia showed the same trend. The seroprevalence of *E. granulosus* was estimated at 8.5%, and the prevalence of infection in dogs reached 23.5% in some regions (M’rad *et al.*, 2021). The control programmes implemented in Tunisia faced different types of constraints that can be divided into categories, as described below.

#### Human constraints

##### Disease perception

As in many parts of the world, perception of this parasitic disease is a cornerstone of both the infection process and prevention programmes. For instance, an integrated programme targeting three zoonotic diseases – CE, leishmaniosis and rabies – coupled with an education programme was implemented in 44 douars (villages) in Morocco. At the end of the programme, the targeted communities remained unconvinced of the need to change some of their risky behaviours [5]. In Tunisia, the perception of CE can be divided into several aspects:

##### Human/dog relationships

In Tunisia, several studies have shown high regional variation in the prevalence of *E. granulosus* infection in dogs ranging from 0% to 23.5% [33]. Human/dog relationships can be classified into two types: (i) a classical or historical relationship where dogs are considered rustic animals that can be fed any low-quality food, such as kitchen waste, old bread, etc., sometimes in insufficient quantities. In rural regions, dogs are also fed raw meat from animal carcasses. Health care is limited to rabies vaccination, since this is offered free of charge by the veterinary services. A large proportion of these animals never receive any veterinary care, and some of them, semi-strays, can even not be caught by their owners. They spend the whole day wandering around the house and only come inside at night to be fed and sleep; (ii) a new relationship between humans and dogs where the animals receive full attention, good food and adequate care. In principle, this category of dogs cannot be involved in the lifecycle of *E. granulosus*. These dogs are properly fed (quantitatively and qualitatively), their food and activity are controlled, and they are properly dewormed.

##### Lifecycle knowledge

Although most Tunisians are aware of CE, many still confuse the species with other parasites or have misconceptions about its life cycle. For example, most Tunisians believe that cat hairs transmit CE “cysts”, more or less confusing them with toxoplasmosis. They also often think that infected offal transmits CE to humans. These confusions limit the effectiveness of any preventive measures taken by the people themselves.

##### Perception of butchers and non-controlled restaurants

**Figure 2:**
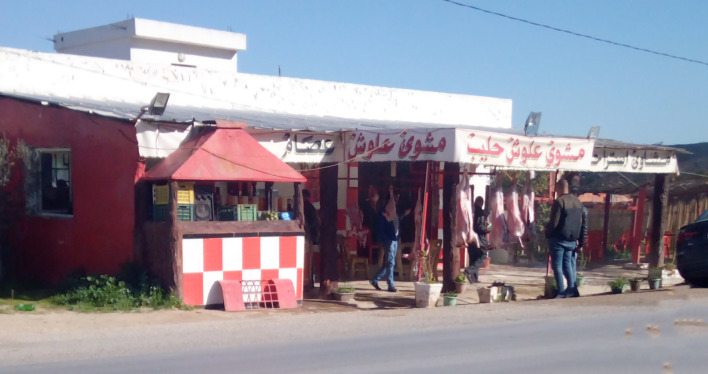
Restaurant preparing grilled meat with non-controlled lamb meat.

One of the social constraints that plays an important role in maintaining the epidemiological cycle of CE is the presence of several road-side grills between Tunisian towns. The staff at these grills slaughter small ruminants (mainly sheep and sometimes goats) and offer travellers grilled lamb meat from non-controlled slaughtered animals (Figure 2). In addition, road-side butchers sell lamb meat of animals slaughtered on the spot without veterinary inspection. A Tunisian study reported that more than 80% of these butchers have a place near the butchery where they slaughter their animals [6]. Both road-side grills and butcher shops are appreciated by Tunisian consumers because the meat is considered fresher.

##### “Willingness to pay” of dog owners in Tunisia

In animal health economics, a distinction needs to be made between willingness to pay and financial capacity. Willingness to pay is the amount of money an economic agent is willing to pay, whereas financial capacity is the amount of money that an economic agent is able to pay. To control CE, dog owners should provide treatment for their animals regularly and continuously (every 6 weeks). This has a significant cost for most people, especially if the dog is large and/or the owner has several dogs. The willingness to pay is low for many dog owners in Tunisia because they see no benefit in treating their dogs, as the parasite is microscopic and harmless to their dogs.

#### Financial constraints: the cost of dog treatment

The only specific cestodicide currently available in Tunisia for dogs is praziquantel, which is combined with oxantel and pyrantel. Praziquantel should be prescribed to dogs at 5 mg/kg every 6 weeks. For example, if an owner has a 30 kg German shepherd, the annual cost of treatment ranges between 124 and 132 Tunisian dinars (corresponding approximately to € 38 and 42), depending on the formulation used. This represents between 2.5% and 2.7% of the Tunisian annual guaranteed agricultural wage.

If we add the cost of ectoparasite treatments, vaccinating dogs against the most common infectious diseases (rabies, distemper, leptospirosis, infectious canine hepatitis and parvovirus disease), veterinary fees, and food costs, owning a dog is very expensive. The first costs to be withdrawn are intuitively the seemingly pointless expense of worming dogs, as *E. granulosus* infection is asymptomatic and has no apparent effect on dog health.

#### High dog populations

Following the Tunisian revolution of 2010, the dog population in the country increased dramatically due to two factors: a decrease in domestic garbage collection and worsening security in both urban and rural areas. The availability of domestic waste food (which may contain offal from animals infected with various parasites, e.g., *Cysticercus tenuicollis*, the larval stage of *Taenia hydatigena*, *Sarcocystis* spp., etc.) for stray dogs and the absence of a stray dog population control programme led to a significant increase in the stray dog population. Their number is in fact estimated at 67,000, with dogs forming large packs on the streets [38]. In addition, the general feeling of insecurity caused by several terrorist attacks and the social problems following the 2010 revolution have led many Tunisians to acquire a dog for the first time. Some of them have been disappointed to discover that these animals are cumbersome and require constant care, and large dogs are difficult to keep in apartments. Some of these new pet owners decided to abandon the dogs, which has increased the problem of stray animals [36]. The infection rate of stray dogs with *E. granulosus* is high and was estimated at between 5% and 16% of the animals tested [26]. As a comparison, the infection rate in Algeria can reach 18.3% (22/120) in Constantine Wilaya (north-east Algeria) [24].

#### Natural constraints

##### Natural borders

The mountainous borders in the northwest of Tunisia and the desert at the southwest border with Algeria (965 km) and Libya in the southeast (459 km) all represent very large areas where it is impossible to control the movements of stray dogs*.* For this reason, there is continuous exchange of infected animals across the borders with these two countries.

##### Biological specificities of the parasite that make it difficult to control

Adults of *E. granulosus* have a lifespan that can reach 2 years, with each parasite shedding approximately one segment every two weeks that remains infective for several weeks in the environment under favourable humidity conditions. However, cysts that are present in intermediate hosts remain infective for several years, which could explain a “bridge effect” with the persistence of *E. granulosus s.l.* even in dry areas [31]. This means that dogs should be treated regularly and *ad eternum* every 6 weeks, which represents a high ongoing cost for dog owners. Finally, infection of both intermediate and definitive hosts is mostly asymptomatic, and when present, symptoms are not specific of *E. granulosus s.l.* infection.

#### Structural constraints

##### Absence of a safe disposal system for dead animals in Tunisia

There is no safe disposal system for dead animals in Tunisia. Bodies and remains are disposed of by the owners themselves. Some animals, such as pets, are most commonly buried. Large or medium-sized animals (dromedaries, cattle, horses, sheep and goats), are usually left where they die (e.g. in pastures), providing infected food for various scavengers, including dogs and other canids.

##### State of Tunisian slaughterhouses

Out of a total of about 200 Tunisian slaughterhouses, only 10 are well equipped [35], and this has a direct negative impact on CE control:

- Free access of animals (dogs and cats) to condemned offal still present in the slaughterhouse (Figure 3).
  **Figure 3:**
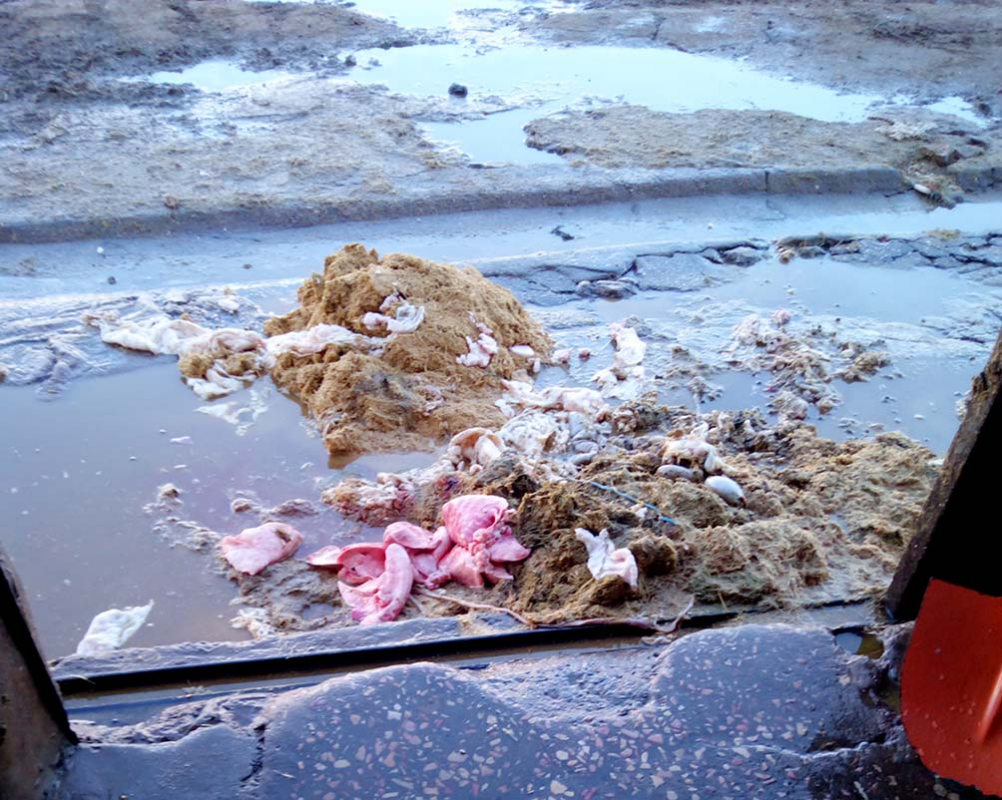
Seized offal in a Tunisian slaughterhouse that is often thrown on the ground.
- The lack of any specific treatment for condemned offal and carcasses, which are thrown directly into public discharges and then left accessible to various stray animals, dogs, cats and wild canids.

In addition, a large number of animals are not slaughtered in slaughterhouses but at home for various festivities (Table 1). In Tunisia, more than half (56.7%) of clandestine butchers throw infected viscera directly into rubbish bins or rivers [6]. In a knowledge, Attitudes and Perception survey performed in Morocco, El Berbri *et al.* [4] reported that 54.3% and 20.4% of infected organs are fed to dogs and dumped in refuse, respectively. Only 19.4% of respondents said that they bury these organs.

**Table 1:** Ranking of slaughter events when slaughtering can be done outside slaughterhouses in Tunisia.

Type of slaughtering	Signification	Frequency	Risk
Muslim feast of slaughtering (*Eid al-Adha*)	An annual Muslim feast, each family slaughter one ruminant, preferably a sheep and in Southern Tunisia (Djerba Island) a bull calf	Yearly, 2 months after Ramadan 1 animal per family	Low since generally the slaughtered animals are young
Weddings	Slaughtering an animal (sheep or bull calf) during the wedding	This practice is less frequent in large towns	Generally low because only young animals are slaughtered
Circumcisions	Slaughtering a sheep during the circumcision	This practice is less frequent in large towns	Generally low because only young animals are slaughtered
Zerda	Slaughtering one ruminant, generally a sheep in marabouts	Yearly	Variable
Guessama (literal translation: sharing)	Practiced by sheep owners, sharing meat of diseased, culled or accidented animals	Occasional; late spring-early summer for culled animals	High, generally animals are aged
Non-controlled restaurants	Restaurants on major road axes	Continuous	Variable, there is a mix of young and aged animals
Non-controlled butcheries	Butcheries in rural and peri-urban regions	Continuous	Variable, there is a mix of young and aged animals

##### Tunisian animal farm typology

Food animal farms in Tunisia are traditionally run with very simple and even archaic infrastructure. Small ruminants are housed in very simple sheepfolds called “guricha”. Walls are often constructed with piles of dried branches. As a result, contact between dogs (shepherd, guard and stray dogs) and ruminants is high and continuous, during the day in the pastures and at night in the sheepfolds.

##### Management of household waste

Due to the high temperatures, especially in summer, domestic waste collection in Tunisia should be daily. After the Tunisian revolution in 2010, frequent social movements and subsequent economic crises negatively impacted the periodicity of household waste collection, providing an important food source for stray dogs, leading to a dramatic increase in their populations and frequent access to *E. granulosus* infected offal.

### One Health and the control of cystic echinococcosis in the Tunisian context

Despite the efforts made by various stakeholders in Tunisia, there are still serious problems associated with CE in both humans and domestic animals, probably with high prevalence in both categories. Although successes in CE control have been recognised, for example in Tasmania and New Zealand, and after the introduction of praziquantel, control efforts have failed almost everywhere [13, 28]: problems with running programmes for long enough (decades), issues with funding, with giving praziquantel to dogs often enough, willingness to vaccinate sheep/dogs on a large scale, working in remote communities with poor infrastructure, problems with stray dogs and an inability to control them (technical feasibility and social acceptability) (Marshall Lightowels, pers. comm.). The multifactorial nature of transmission factors has been recognised by some authors and this is also the case for Tunisia [10]. Furthermore, the weight of each factor may vary depending on local circumstances. This is a key point to consider when implementing control programmes.

**Figure 4:**
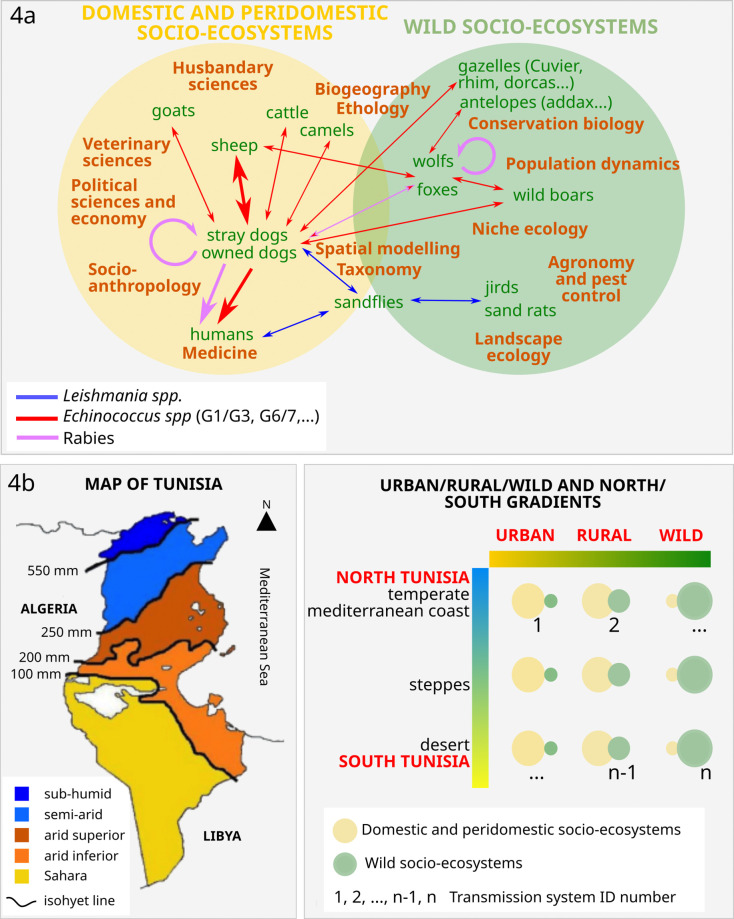
General epidemiology of the three main zoonotic diseases involving dogs in Tunisia: cystic echinococcosis (*Echinococcus granulosus* infection), leishmaniosis (*Leishmania infantum* infection) and rabies. (a) Transmission routes of *Echinococcus* spp., *Leishmania* spp. and rabies in Tunisia (domestic and peri-domestic and wild socio-ecosystems) and the disciplines involved in their control. The yellow circle limits the space of domestic and peri-domestic transmission, the green circle the space for wildlife transmission. The figure shows how different disciplines (in brown) articulate and can contribute to knowledge and control of these three zoonotic diseases involving dogs. (b) Based on the geography of Tunisia (map after [18]), we hypothesise the existence of different patterns of transmission of the three zoonotic diseases involving dogs with “north–south “ and “urban–wild” gradients. The size of each circle is proportional to its importance in the transmission system. Along each gradient, the contributions of hosts and pathogens and the weights of the driving factors vary, possibly leading to different transmission patterns (numbered 1, 2, ..., *n* − 1, *n*).

The WHO, WOAH, FAO and UNEP have jointly updated the concept of One Health, with the intention of translating this concept into policies and concrete actions [1]. This may apply to *E. granulosus* transmission in Tunisia. The approach mobilises multiple sectors, disciplines, and communities at varying levels of society to work together to foster well-being and tackle threats to health and ecosystems. For those reasons, CE control programmes should take into account specificities at various scales and be based on a sound knowledge of the local context in which they are implemented. Furthermore, economies of scales can be achieved considering a number of diseases transmitted by dogs in the same programme. Figure 4 shows how climate and socio-ecosystems and possible local differences should be considered for a national programme with local specificities. It shows that lifecycles and contexts should be considered differently according to “north–south” and “urban–rural” gradients. It also shows how several sectors and disciplines should articulate to understand and capture the whole transmission system locally.

Since, in general, no single control measure is 100% effective in multifactorial socio-ecosystems, the control of CE calls for the concomitant implementation of several control options, targeting definitive hosts (to protect the intermediate host) and intermediate hosts (to reduce the risk of infection of definitive hosts).

General recommendations about CE control have been published in several articles (see e.g., [13] for a synthesis). Here we present a table summarising the control measures applicable in Tunisia in relation to the scales of feasibility in the Tunisian context (Table 2).

**Table 2:** Control measures applicable in Tunisia associated with scales of feasibility in the country’s context (1 low feasibility, 6 high feasibility).

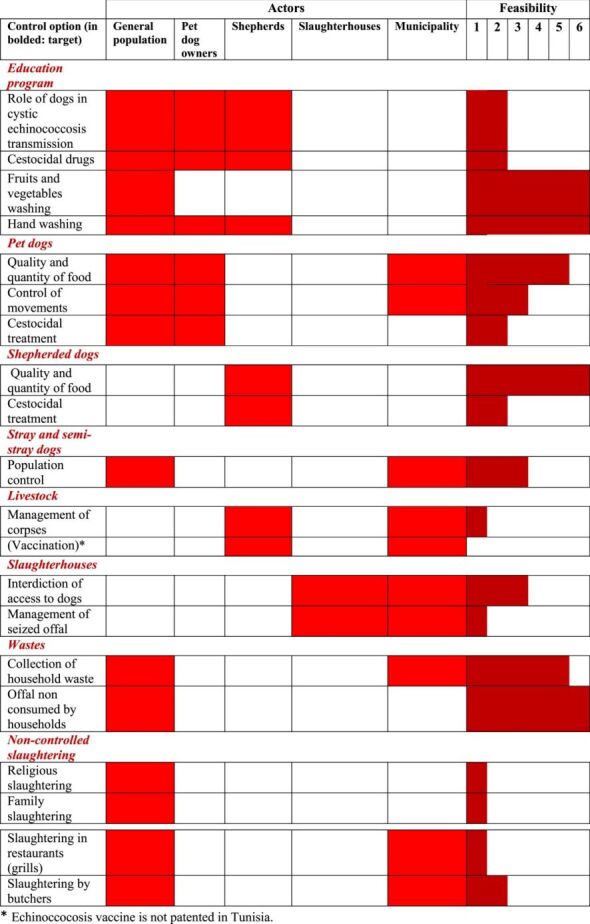

**Figure 5:**
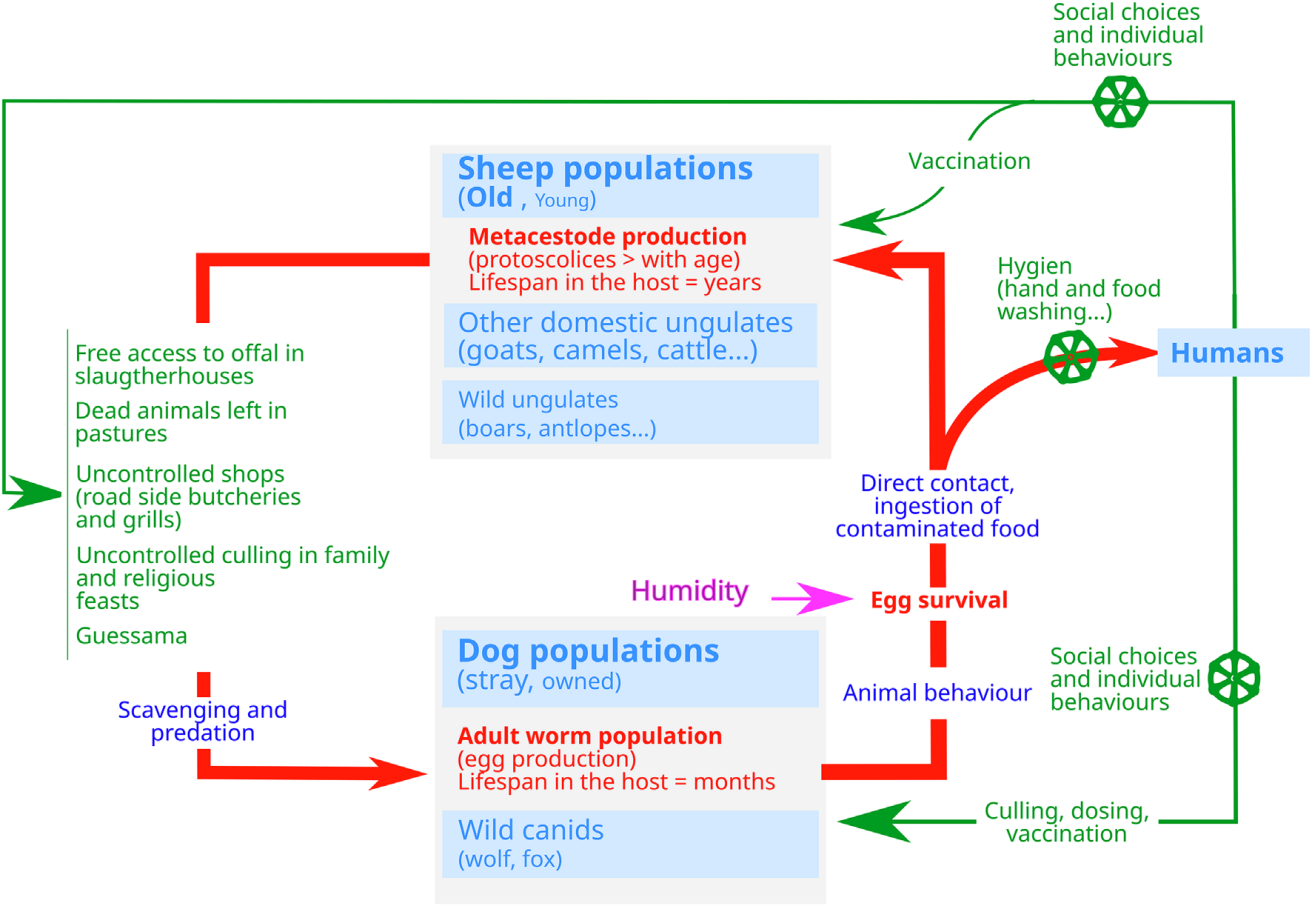
General patterns of transmission of *Echinococcus* spp. and control points in Tunisia. In red, the parasite route; in blue, the hosts involved and behaviours favouring transmission (the size of the characters indicates their presumed importance in transmission); in green, human actions that may modulate transmission.

On this basis, Figure 5 summarises the pattern of transmission and control points for Tunisia.

Multiple collaborations with researchers of relevant disciplines including sociologists, sectors such as human health, animal health and, where appropriate, wildlife ecologists, representatives of stakeholders and civil society involved since the outset, etc., close to and adapted to the specificities of local situations should be strongly encouraged so that programmes can be sustained. This requires public acceptance and support over the long term of several decades with regular evaluation of their impacts.

Below we outline a number of key issues in Tunisia where multidisciplinary collaborations with stakeholders would help to remove barriers to CE control.

#### Control of dog infection

Individual dogs and categories (stray dogs, shepherd dogs, pet dogs, etc.) may exhibit very different behaviours resulting in different epidemiological patterns of *E. granulosus* transmission. If dogs are consistently given safe and sufficient food and are supervised and kept at home or leashed, they are unlikely to scavenge. Dogs that roam, or dogs whose feeding cannot be controlled, or that voluntarily feed on uncontrolled offal, should be treated with praziquantel at adequate doses (5 mg/kg) on permanent and regular basis. The effectiveness of dog treatments depends on the frequency of treatment (every 1.5 month is recommended, roughly the duration of the prepatent period), a frequency much greater than the “twice a year” generally recommended in Tunisia [25].

A control programme for CE in Morocco consisting of treatment of owned dogs on a 4-monthly basis did not induce a reduction in the level of the parasite transmission [2].

Reducing the cost of treatments for dogs by subsidizing drugs and increasing the awareness of dog owners through targeted information campaigns could help improve the effectiveness of treatment programmes. These measures can be combined with individual identification (e.g., tattoo or transducer, the former being cheaper), and vaccination against rabies. Furthermore, as culling is not ethically acceptable, the stray dog population should be reduced by sterilising both males and females until they are removed from the streets. In Tunis, a veterinarian has vaccinated against rabies and sterilised approximately 2,500 dogs in two years. He confirmed that “without a massive sterilisation campaign, the streets will be invaded by stray dogs” [29]. The potential for effective vaccination of dogs against *E. granulosus* is still uncertain at this time [28].

#### Infection control in livestock

Ruminants are the main intermediate hosts of *E. granulosus sensu stricto* in North Africa, and limiting their infection is difficult. Dogs and other canids shed parasite eggs in pastures, and they can be transported at long distances by runoff water and coprophagous insects [31].

In addition, on farms, dogs are often kept in the same area as sheep and cattle, where dog faeces are concentrated and the risk of infection is high.

Cysts are very resistant and remain fertile and infective in intermediate hosts for several years. When ruminants die on pastures, there is no legal requirement for the owner to bury the carcass. Left in place, they are eaten by various scavengers including dogs. Changing this practice is very difficult for a number of reasons.

- Animals die every day across the country and some regions are very remote with no infrastructure.
- The cost of safe disposal of animal carcasses, especially large animals, is too high and cannot generally be afforded.
- Last but not least, the mentality of livestock owners can make them reluctant to change traditional habits and personal feelings. MG has personally necropsied an ewe in the pasture and explained to the owner that it had died of severe bronchopneumonia with dozens of *Echinococcus* cysts in the lungs. When he asked the farmer to bury it, after explaining the risk of transmission, he was told that the body was too far from the farm (approximatively 200 m from the main gate).

Vaccination of intermediate hosts of *E. granulosus* with the recombinant vaccine EG95 can be used to reduce the level of parasite transmission. Several programmes in South America, China and Morocco have shown that vaccination schemes involving two injections in lambs and an additional injection at approximately one year of age are effective in reducing cyst numbers and fertility. Although sheep vaccination requires fewer interventions per year compared to dog treatment, it also requires more infrastructure and must be completed in a short period of time to minimise costs, and it involves a higher number of animals compared to dog dosing [2, 15, 28]. A trial is currently underway in Tunisia as part of the ECHINO-SAFE-MED programme of the EU Funded PRIMA partnership (https://www.echinosafemed.com).

#### Control of vegetable contamination

Fresh vegetables are contaminated by *E. granulosus* eggs either in vegetable gardens (where dogs generally have access) or in vegetable shops (where stray dogs have access to markets, especially at night). For example, [34] found 1.25% contamination in fresh vegetables sold for human consumption. Vegetable gardens and markets should be made inaccessible to dogs. Moreover, fresh vegetables should also be protected from insects and stray dogs.

#### Waste management

Waste has twofold importance, (i) it is a source of parasites for dogs, where they can find parts of organs with cysts left behind by people (e.g., home slaughter) or even professionals from slaughterhouses; (ii) it is a source of food for stray dogs, which leads to an increase in their population and their constant displacement in search of food. Waste should be collected daily to reduce its role in the epidemiological cycle of CE.

#### Slaughter management

Slaughterhouse buildings are generally not protected from animals, including dogs and cats, and their refurbishment, which is costly, is a major challenge in Tunisia. In addition, offal is not destroyed, remains accessible, and is sometimes fed directly to dogs.

In Tunisia, as in almost all developing countries, non-controlled slaughter is common and occurs on various occasions and in various contexts, including religious, family and traditional celebrations (see Table 1). This kind of slaughter is not controlled and is generally carried out by unqualified persons. Specific health inspection of slaughtered animals could be introduced, adapted to the different Tunisian contexts, in order to increase the proportion of slaughtered animal inspected. Most of the control efforts could be focused on old sheep, as there is a positive correlation between age and prevalence [25].

As long as the reasons for home slaughtering are different between social groups and people, sociological studies could help to improve the involvement of different stakeholders and the acceptance of control programmes adapted to the respective objectives and attitudes. The “Aïd without cyst” campaign is an example of a programme run in Tunisia since the 1980s by the National Association of Tunisian Veterinarians. It involves dozens of volunteer veterinarians who make themselves available, physically or by telephone, during Aïd-El-Kebir, a Muslim festival where a sheep is sacrificed and distributed to family and friends. Information and the list of volunteers are published a few days in advance in the media (newspapers, lists in the various official administrations, and now on Meta (formerly Facebook) and the internet, etc.). Citizens who notice an abnormality in their slaughtered animals are invited to either bring the suspect organ to one of the centres or call one of the volunteers. This campaign has been and continues to be a great success throughout Tunisia.

#### Educational programmes

The well-known and ultimately successful control programme in Iceland started in the 19th century, lasted for decades, and eliminated transmission from the island by the 1950s–1960s. Craig *et al.* [13] note that “this was in many ways a unique situation where a small, highly literate population responded positively to health education messages and legislation to stop home slaughter, reduce dog contacts and accept annual arecoline treatment of dogs”. Most other educational campaigns in other countries did not appear to have a significant impact on *E. granulosus* transmission. Faster-track vertical programmes based on regular dog dosing have been key to the success of most hydatid control programmes, although success was partial and transient due to lack of continuity. It is clear that the success of control programmes depends on understanding and acceptance by target populations. This depends on knowledge about how the general public and decision-makers accept to move from knowledge to action, individually and collectively. For instance, anthropologists studying the perception of the risk of alveolar echinococcosis (*Echinococcus multilocularis* infection) in a farming population of Franche-Comté, France showed that behavioural changes depend on how farmers individually represent the risk, and that actual behaviour change depends on individual history (e.g., proximity to a family member or friend affected by the disease, etc.) [22]. Sociologists have also shown that people are generally reluctant to change their behaviour in response to a hazard perceived as distant in time and virtually invisible, and that they need to face an immediate danger or have an immediate and perceptible benefit to change. Anthropological studies, with the involvement of local stakeholders and communities, are highly recommended for the effectiveness of educational programmes on CE control.

### Practical way forward for a cystic echinococcosis control programme in Tunisia

The practical aspects of implementing a control programme targeting CE are the main cornerstone for the programme. In fact, Tunisia has the various prerequisites for success of the programme, in particular human resources (animal and human health specialists at various levels), a good administrative organisation and collaboration between the human and animal health services, and a relatively good health network covering most of the county. A control programme could take into account the following key points:

#### General organisation of a CE control programme

One of the key pillars for this control programme is the adoption of a One Health approach. For this reason, several ministries could be involved in the programme: Ministry of Agriculture (treatment of dogs, control of ruminant populations), Ministry of the Interior (management of slaughterhouses, control of dog populations and domestic waste collection), Ministry of the Environment (management of the environment in close collaboration with the Ministry of the Interior, actions on the wildlife component, where appropriate), Ministry of Higher Education and Research (basic and applied research programmes, educational programmes targeted to new school teachers and continuing education, etc.) and Ministry of Health (management of the human clinical cases, notification of human cases). All these ministries could also work together to set up professional training and education programmes. Both actions could be carried out within a joint commission involving all the ministries. The same commission could periodically communicate the results of different actions to all stakeholders and to the Tunisian population in general.

#### Funding for a cystic echinococcosis control programme

This is one of the most important limitations of any control programme, especially the sustainability of funding. One of the solutions to reduce the cost of the control programme is to link it to other diseases as an integrated control programme of zoonotic diseases in dogs. In Tunisia, three important diseases could be linked, namely CE, leishmanioses and rabies. This association will certainly reduce the cost of each programme but will also involve a greater number of stakeholders as was successfully done in Sidi Kacem Province (northwestern Morocco), where 22 control and 22 treated douars (villages) were included in an integrated programme targeting leishmaniosis, rabies and CE [5]. To increase the acceptance and compliance of any control programme, a participatory approach could be adopted from the early stages of its establishment. In practice, non-governmental organisations (NGOs) and farmers should be actively involved in CE control. Although we acknowledge that health socio-economists might provide more insights into the economic aspects, we suggest that these two stakeholders could be involved in funding either through direct collection of funds or through the introduction of a specific small tax on lamb meat at slaughterhouses for NGOs and livestock owners, respectively. They represent important and sustainable sources of funding that could achieve the elimination of the infection. Participating Tunisian ministries could contribute at a fixed percentage of the total funds.

A good communication plan could be put in place before and during the implementation of the programme, not only to involve other funders but also to keep a high level of motivation for all the partners.

#### Evaluation of the control programme

Evaluation is an additional cornerstone of any control programme and relevant criteria need to be carefully selected and shared with all stakeholders There are two major threats that could hamper a programme: (i) sometimes, political and social considerations motivate some animal and/or human health stakeholders to change the indicators and make them more optimistic by masking the results. The indicators chosen must therefore be transparent, public and verified by an independent body; (ii) demotivation of the various actors that provide significant investment due to a lack of feedback about the results obtained.

## Conclusion

CE is a major human and animal health problem in all regions of Tunisia. Several attempts to control this parasitic infection through national campaigns have failed because they targeted only one component of the infection (either human or animal), with little or no regard for differences between socio-ecosystems. The One Health approach is the best framework to address all components of this infection simultaneously: human health, animal health and ecosystem health. Taking these elements into account at the same time and for the entire duration of the control programme could dramatically reduce the impact of this parasite on both humans and animals.
